# Electrochemical allylations in a deep eutectic solvent

**DOI:** 10.3762/bjoc.20.189

**Published:** 2024-09-02

**Authors:** Sophia Taylor, Scott T Handy

**Affiliations:** 1 Department of Chemistry, Middle Tennessee State University, Murfreesboro, TN, USA

**Keywords:** allylation, electrosynthesis, eutectic solvent, recycling, tin

## Abstract

Electrosynthesis is a technique that is attracting increased attention and has many appealing features, particularly its potential greenness. At the same time, electrosynthesis requires a solvent and a supporting electrolyte in order for current to pass through the reaction. These are effectively consumable reagents unless a convenient means of recycling can be developed. As part of our interest in unusual solvents and electrochemistry, we explored the application of simple, inexpensive, and recyclable deep eutectic solvents to the allylation of carbonyls. While several sets of conditions were developed, the goal of avoiding stoichiometric amounts of metal has proven elusive. Still, a deep eutectic solvent can be used to plate out and thus recover the metal used, offering an interesting new option for electrochemical allylations.

## Introduction

The last several years have witnessed a tremendous resurgence of interest in electrochemistry in the area of organic synthesis [1]. While there are many reasons for this renewed interest, two major motivations are the unmatched control of oxidation or reduction potential that can be achieved and the environmentally friendly aspect of having electrons as the only consumed reagent. This latter reason is certainly an advantage in many cases, but its effective realization is limited by the need for both a solvent as well as a supporting electrolyte to allow for the flow of current through the reaction. Although some imaginative options have been reported, they tend to be quite limited in scope. Room temperature ionic liquids (RTILs) were viewed as interesting options that would combine both the solvent and the electrolyte into one component and could be readily recycled due to their non-volatile nature [2–15]. Further, many of these species featured very wide electrochemical windows. In practice, however, RTILs are expensive compared to conventional solvents. Most of them are also quite viscous, which severely limits their use in synthetic electrochemistry [16].

These same expense and viscosity issues plague the application of RTILs in any area. Driven by this limitation, deep eutectic solvents (DES) were reported and have been heavily promoted as cost-effective replacements for RTILs in many applications [17–18]. One of the early and very effective applications of DES was in the area of electroplating as well as metal recovery by electrodeposition [19]. Despite this fascinating potential, very little has been reported in terms of their use in electrosynthesis [20–24].

Given our interest in both electrosynthesis and DES, we opted to explore this combination in the area of electrochemical allylation. The allylation of carbonyls is a valuable reaction that has been explored under a wide range of conditions, including several reports using electrochemical conditions [25–26]. Recently, an allylation in DES has also been reported, but using indium as the reducing metal under standard Barbier-type conditions, but not electrochemical ones [27]. While the unusual DES (acetylcholine chloride/acetamide 2:1 molar ratio with added ammonium chloride) could be recycled five times with a modest decrease in yield (99% to 65%), the requirement of using suprastoichiometric amounts of expensive indium metal for each reaction is a significant drawback. The potential to avoid this limitation by using electrochemistry with catalytic amounts of metal or perhaps by recovering and recycling the metal with a recyclable solvent was the goal motivating this project. With this as a background, we undertook an investigation of electrochemical allylation in DES.

## Results and Discussion

While there are many potential DES that are known, one that would be stable to highly reducing conditions was desired for the allylation reaction. Further, to make the reaction conditions similar to standard electrochemical ones, a solvent with a tetraalkylammonium salt, the first DES that was studied was the 1:3 molar ratio of tetrabutylammonium bromide and ethylene glycol (TBAB/EG) [28]. Using the reaction of *p*-anisaldehyde with allyl bromide as a test case, reactions were performed using three sets of different sacrificial electrodes as well as non-sacrificial graphite. As can be seen in Table 1, tin (entry 1) resulted in good conversion to the allylation product, while zinc, magnesium, and graphite (Table 1, entries 3–5) displayed no reaction at all. This observation was somewhat surprising considering that both zinc and tin are very commonly used in conventional allylations in addition to as electrode materials for electrochemical allylations [29–48]. Further optimization employing tin (Table 1, entry 2) enabled near complete conversion to the desired product by using 2.5 F/mol of current passed through the solution at a constant current of 20 mA.

**Table 1:** Influence of electrode material.

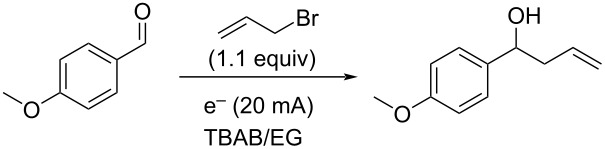			

Entry	Electrode	F/mol	% Conversion

1	Sn/Sn	2	86
2	Sn/Sn	2.5	91^a^
3	Zn/Zn	2.5	0
4	Mg/Mg	2.5	0
5	C/C	2.5	0

^a^Isolated yield of product 78%.

With this promising start, several other aldehydes and two ketones were explored under the same reaction conditions (Table 2). A range of aromatic aldehydes worked well as well as one aliphatic aldehyde (Table 2, entry 13) and one alkenyl aldehyde (Table 2, entry 10), although the highly electron-rich dimethylaminobenzaldehyde (Table 2, entry 4) afforded only recovered starting material. Both ketones (Table 2, entries 11 and 12) failed to react and resulted in just recovered starting material. 4-Nitrobenzaldehyde (Table 2, entry 5) also failed to afford any allylation product, although in this case, reduction of the nitro group to an amino group was observed and the resulting 4-aminobenzaldehyde is likely too electron-rich to undergo allylation.

**Table 2:** Aldehyde variations.

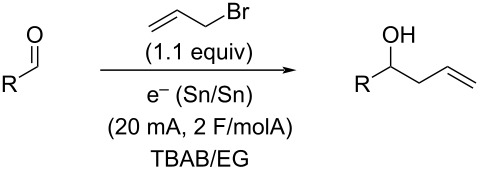		

Entry	R	Isolated yield

1	*p*-MeOC_6_H_4_	78%
2	*p*-BrC_6_H_4_	82%
3	*p*-CNC_6_H_4_	80%
4	*p*-Me_2_NC_6_H_4_	NR
5	*p*-NO_2_C_6_H_4_	0%^a^
6	*p*-MeC_6_H_4_	85%
7	*m*-MeC_6_H_4_	79%
8	*o*-MeC_6_H_4_	76%
9	Ph	68%
10	cinnamyl	77%
11	acetophenone	NR
12	cyclohexanone	NR
13	C_6_H_11_	84%

^a^Only nitro group reduced, no allylation.

The use of other halides was also explored (Table 3). Switching to allyl chloride (Table 3, entry 2) did result in partial conversion, but the reaction was much less efficient than for allyl bromide. More substituted allyl bromides, such as crotyl and prenyl bromide (Table 3, entries 3 and 4) did react, although they afforded only partial conversion when using 2.5 F/mol of current. In terms of regiochemistry, addition at the more substituted end (gamma addition) was the major product in both cases, although alpha addition was also observed. In the case of crotyl bromide, no diastereoselectivity was noted for the gamma product. Non-allylic halides such as benzyl bromide, propargyl bromide, and ethyl bromoacetate (Table 3, entries 5–7) did not result in carbonyl addition. The benzyl bromide was recovered intact, while for the other two, they are both sufficiently volatile that they would have been lost during the work-up. For all three, the aldehyde was recovered unreacted.

**Table 3:** Halide variations.

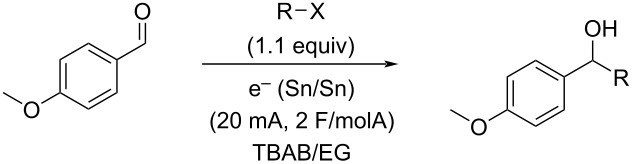		

Entry	R–X	% Conversion^a^

1	allyl bromide	100%
2	allyl chloride	50%
3	crotyl bromide	85%^b^
4	prenyl bromide	45%^c^
5	ethyl bromoacetate	NR
6	benzyl bromide	NR
7	propargyl bromide	NR

^a^Determined by ^1^H NMR of the crude extract; ^b^γ to α addition 2.2:1.0, 1:1 diastereomeric mixture of the γ product; ^c^γ to α addition 3:1.

While the use of DES as solvent and electrolyte for electrochemical allylation had been demonstrated, there was no particular advantage to this method over non-DES options without recycling of the DES. To explore this aspect, the allylation of anisaldehyde with allyl bromide was undertaken using TBAB/EG. As can be seen in Table 4, the DES could be recycled two times before incomplete conversion was noted. A more significant problem was the steady loss of DES during the product extraction stage. Using 3 mL of methoxycyclopentane in a single extraction, the amount of DES steadily decreased by roughly 0.5 mL for each recycling. This is likely due to partial solubility of ethylene glycol in methoxycyclopentane as ethylene glycol could be clearly seen in the ^1^H NMR spectrum of the crude reaction extracts. It should also be noted that the lower isolated yields most likely reflect mechanical losses during extraction and chromatographic separation as the crude spectra do not show significant amounts of side products.

**Table 4:** Recycling for the allylation of *p*-anisaldehyde in TBAB/EG DES and Sn electrodes.

Cycle	Volume of DES recovered	Conversion/isolated yield^a^

1	2.5 mL	100%/78%
2	2.0 mL	100%/77%
3	1.5 mL	100%/79%
4	1.25 mL	80%/60%

^a^Determined by ^1^H NMR of the crude extract.

Another problem that was noted in these recycling experiments was a build-up of tin salt byproducts that made the DES more and more viscous in each recycling. Avoiding the use of a sacrificial electrode could help this aspect as well as reducing the waste generated in these allylations. While non-sacrificial graphite electrodes had failed to result in any reaction, it seemed possible that the use of a catalytic amount of tin metal or a tin salt with graphite electrodes would result in a superior reaction due to in situ reduction and/or activation of the tin. This variation was explored as seen in Table 5. Use of 0.5 equivalents of tin metal (Table 5, entry 1) did result in partial conversion to product, which increased to complete conversion when 1.5 equivalents of tin were used (Table 5, entry 3). Upon attempted recycling of this reaction, very little conversion was noted, indicating that this method simply exchanged one form of consumed tin (the electrode) for another (the metal powder). We also explored the use of SnCl_2_ as the tin source. Little to no reaction occurred under a variety of constant current conditions (Table 5, entries 5 and 6), but using two equivalents at a constant potential of 2.0 V (Table 5, entry 8) resulted in complete conversion to the allylation product. As before, attempts to directly recycle the salt/DES mixture failed to afford any allylation.

**Table 5:** Tin metal with non-sacrificial electrodes.

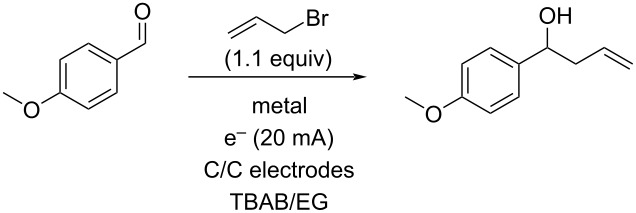			

Entry	Tin source	Equivalents of tin	% Conversion^a^

1	Sn powder	0.5	15%
2	Sn powder	1	40%
3	Sn powder	1.5	100%
4	SnCl_2_	1	10%
5	SnCl_2_	2^b^	NR
6	SnCl_2_	2^c^	NR
7	SnCl_2_	2^d^	50%
8	SnCl_2_	2^e^	100%

^a^Determined by ^1^H NMR of the crude extract; ^b^100 mA constant current; ^c^50 mA constant current; ^d^100 mA constant current and 10% by volume water added; ^e^constant potential of 2 V.

Next, it was decided to compare the results of the sacrificial tin electrodes at a constant current of 20 mA with the non-sacrificial glassy carbon electrodes and tin(II) chloride at a constant potential of 2 V for a set of aldehydes. As can be seen in Table 6, three of the four cases (entries 1, 2, and 4), the glassy carbon conditions afforded better yields of the allylation product. Only in the case of the cyclohexanecarboxaldehyde (Table 6, entry 3) was the yield lower and in this case this lower yield was the result of incomplete conversion of the aldehyde.

**Table 6:** Electrode comparison in TBAB/EG.

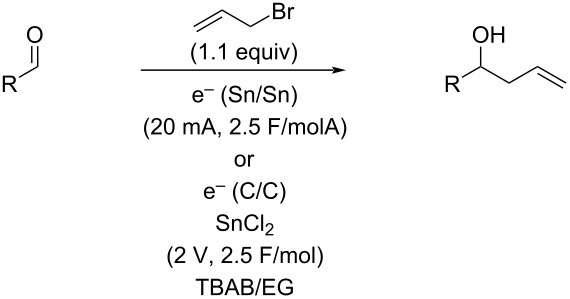			

Entry	Aldehyde	Sn/Sn isolated yield	C/C with SnCl_2_ isolated yield

1	*p*-anisaldehyde	83%	91%
2	*p*-tolualdehyde	77%	83%
3	cyclohexane- carboxaldehyde	68%	58%^a^
4	cinnamaldehyde	61%	85%

^a^12% recovered aldehyde.

For as successful as the TBAB/EG conditions were, TBAB is not inexpensive. Choline chloride is much less expensive and is particularly well known and frequently used in DESs. It is known that choline chloride and ethylene glycol will also form a DES when combined in a 1:2 molar ratio [49]. This solvent is much more viscous than the TBAB/EG one, and an initial attempt to use the same 20 mA constant current conditions with sacrificial tin electrodes resulted in 50% conversion to the allylation product and starting material after passing 2.5 F/mol of current. This poor conversion could be significantly improved by adding 10 volume percent of water to the reaction mixture, which also visibly decreased the viscosity. While complete conversion was still not achieved (67% conversion), it was a considerable improvement. Using glassy carbon electrodes with two equivalents of tin(II) chloride under a constant potential of 2 V, but still in CC/EG with 10 volume percent water, resulted in further improvement (75% conversion) for the same reaction with anisaldehyde. Comparing these two variations always resulted in improved conversions and yields for the glassy carbon conditions, although the results were generally not superior to those obtained in TBAB/EG (Table 7). Interestingly cyclohexanecarboxaldehyde (Table 7, entry 3) was again the outlier as it afforded an excellent yield and complete conversion in the case of glassy carbon in CC/EG.

**Table 7:** Electrode comparison in CC/EG.

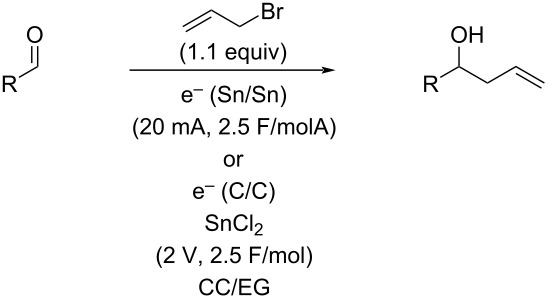			

Entry	Aldehyde	Sn/Sn isolated yield	C/C with SnCl_2_ isolated yield

1	*p*-anisaldehyde	34%^a^	62%^b^
2	*p*-tolualdehyde	24%^b^	67%^c^
3	cyclohexane- carboxaldehyde	52%^c^	96%
4	cinnamaldehyde	58%^d^	62%^b^

^a^Reaction 50% completion; ^b^reaction 75% completion; ^c^reaction 67% completion; ^d^reaction 80% completion.

While the CC/EG system did not appear in general to be as successful as the TBAB/EG one in terms of yields and conversions, it was decided to explore DES recycling (Table 8). As with TBAB/EG, the allylation of *p*-anisaldehyde with allyl bromide was conducted using 2 equivalents of tin(II) chloride in CC/EG with 10 volume percent water at a constant potential of 2 V until 2.5 F/mol of current had been passed through the reaction. The product and unreacted starting material were recovered via extraction with methoxycyclopentane and the CC/EG used in another cycle. Through two recyclings, the reactions were similar in efficiency, although the DES became increasingly viscous as the tin byproducts built up and it became impractical to recycle the DES further.

**Table 8:** Recycling in CC/EG.

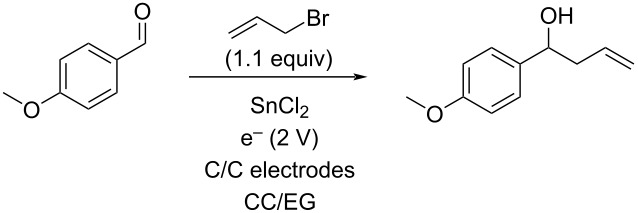	

Cycle	Isolated yield

1	64%
2	68%
3	72%

While the DES clearly had some ability to be recycled, the use of catalytic amounts of metal had uniformly failed. Recognizing that DES have been used extensively for electroplating and metal deposition, though it seemed that this might provide an opportunity for metal recovery and reuse [19]. To this end, the DES mixture from cycle 3 of Table 8 following product extraction was electrolyzed at 100 mA constant current using glassy carbon electrodes until 2 F/mol of current had passed. This deposited a metal clump on the electrode that was removed and then analyzed using X-ray fluorescent spectroscopy to determine that the deposited metal was tin, with traces of other metals consistent with the purity of the initial tin source. The tin recovery was 99% of the theoretical. To explore recycling of this recovered tin, 1 mmol was used in a 0.5 mmol scale allylation of anisaldehyde under the constant potential conditions with glassy carbon electrodes and fresh DES. This reaction afforded the anticipated product in 74% yield, thus demonstrating that the tin can be recovered and recycled.

## Conclusion

In conclusion, we have demonstrated the ability to use DES as a combined solvent and electrolyte for electrosynthesis. It can be recycled, although product extraction does result in a slow but detectible loss of the ethylene glycol. Attempts to use catalytic amounts of metal with non-sacrificial electrodes in place of the sacrificial tin electrodes were not successful, but we were able to demonstrate efficient and near quantitative recovery of the metal by electrolysis after product extraction and this recovered metal can be used again for further allylations. Further efforts to improve the efficiency and enable catalytic metal use are underway.

## Experimental

### Preparation of deep eutectic solvents

#### Tetrabutylammonium bromide/ethylene glycol (1:3 molar ratio) deep eutectic solvent

To 8.0 grams of tetrabutylammonium bromide (TBAB) were added 4.7 grams of ethylene glycol (EG). The resulting mixture was heated to 70 °C until a homogeneous liquid formed. It was stored at this same temperature between uses.

#### Choline chloride/ethylene glycol (1:2 molar ratio) deep eutectic solvent

To 6.98 grams of choline chloride (CC) were added 6.2 grams of EG and the resulting mixture was heated to 70 °C until a homogeneous liquid formed. It was stored at this same temperature between uses.

### General Sn/Sn procedure with TBAB/EG

To a 10 mL ElectraSyn 2.0 vial containing a magnetic stir bar, the carbonyl compound (0.5 mmol), allyl bromide (0.6 mmol), and TBAB/EG DES (2 mL) were added. Tin electrodes were used as the working and counter electrodes and were submerged into the reaction. The reaction was performed under constant current conditions of 20 mA with no reference electrode until 2.5 F/mol was passed. The current was programmed to alternate every five minutes to minimize any potential fouling of the electrode surface. Following completion of each reaction, the mixture was transferred to a separatory funnel with the aid of 10 mL of deionized (DI) water followed by 20 mL of diethyl ether. The organic layer was separated, dried with anhydrous magnesium sulfate, filtered, and the solvent removed in vacuo to afford the crude product, which was first analyzed by ^1^H NMR spectroscopy and then purified using flash column chromatography. In between reactions, the tin electrodes were rinsed with DI water and acetone, then polished using diamond polish. This helped prevent buildup on the electrode surfaces.

### General C/C procedure with TBAB/EG

To a 10 mL ElectraSyn 2.0 vial containing a magnetic stir bar were added the carbonyl compound (0.5 mmol), allyl bromide (0.6 mmol), and TBAB/EG DES (2 mL) along with SnCl_2_ (0.1896 g, 1 mmol). Graphite electrodes were used as the working and counter electrodes and were submerged into the reaction. The ElectraSyn was programmed to run the reaction under constant potential conditions of 2 V with no reference electrode until 2.5 F/mol was passed. Following completion of each reaction, the mixture was transferred to a separatory funnel with the aid of 10 mL of DI water followed by 20 mL of diethyl ether. The organic layer was separated, dried with anhydrous magnesium sulfate, filtered, and the solvent removed in vacuo to afford the crude product, which was first analyzed by ^1^H NMR spectroscopy and then purified using flash column chromatography. The graphite electrodes were not polished in between reactions, but were rinsed with DI water and then acetone.

### General Sn/Sn procedure with CC/EG

To a 10 mL ElectraSyn 2.0 vial containing a magnetic stir bar, the carbonyl compound (0.5 mmol), allyl bromide (0.6 mmol), CC/EG DES (2 mL), and 10% DI water (0.2 mL) were added. Tin electrodes were used as the working and counter electrodes and were submerged into the reaction. The ElectraSyn was programmed to run the reaction under constant current conditions of 20 mA with no reference electrode until 2.5 F/mol was passed. The current was programmed to alternate every five minutes. Following completion of each reaction, the mixture was transferred to a separatory funnel with the aid of 10 mL of DI water followed by 20 mL of diethyl ether. The organic layer was separated, dried with anhydrous magnesium sulfate, filtered, and the solvent removed in vacuo to afford the crude product, which was first analyzed by ^1^H NMR spectroscopy and then purified using flash column chromatography. The electrodes were washed with DI water and acetone before polishing.

### General C/C procedure with CC/EG

To a 10 mL ElectraSyn 2.0 vial containing a magnetic stir bar were added the carbonyl compound (0.5 mmol), allyl bromide (0.6 mmol), CC/EG DES (2 mL), and 10% DI water (0.2 mL) along with SnCl_2_ (0.1896 g, 1 mmol). Graphite electrodes were used as the working and counter electrodes and were submerged into the reaction. The ElectraSyn was programmed to run the reaction under constant potential conditions of 2 V with no reference electrode until 2.5 F/mol was passed. Following completion of each reaction, the mixture was transferred to a separatory funnel with the aid of 10 mL of DI water followed by 20 mL of diethyl ether. The organic layer was separated, dried with anhydrous magnesium sulfate, filtered, and the solvent removed in vacuo to afford the crude product, which was first analyzed by ^1^H NMR spectroscopy and then purified using flash column chromatography. The graphite electrodes were not polished in between reactions, but were rinsed with DI water and then acetone.

### DES and tin metal recycling

For the recyclability trials, the electrolysis vial was opened and about 3 mL of methoxycyclopentane was pipetted directly onto the reaction solution. Then, the vial was capped with a rubber stopper. The methoxycyclopentane acted similarly to the diethyl ether in previous separatory methods by extracting the product from the DES. After letting the methyoxycyclopentane and DES layers separate, the methoxycyclopentane layer was removed using a pipette, placed into a round-bottomed flask, and concentrated in vacuo. This remaining DES could be used directly in subsequent reactions.

If SnCl_2_ was used in the reaction, electrolysis of the tin metal could be achieved after workup with methoxycyclopentane. Once all product was extracted, the graphite electrodes were submerged in the remaining DES solution in the vial and the tin metal (around 3 mmol) was electrolyzed at 100 mA constant current until 2 F/mol had passed. The resulting metal clump on the electrode was removed using a scoopula for further use and analysis using a Thermo Scientific Niton XL3t X-Ray Fluorescent Spectroscopy analyzer.

### Product characterization

**1-(4-Methoxyphenyl)-3-buten-1-ol** [39]: ^1^H NMR (300 MHz, CDCl_3_) 7.27 (d, *J* = 8.58, 2H), 6.86 (d, *J* = 5.82, 2H), 5.83–5.73 (m, 1H), 5.17–5.10 (m, 2H), 4.67 (t, *J* = 6.18, 1H), 3.80 (s, 3H), 2.49 (t, *J* = 4.8, 2H).

**1-(4-Bromophenyl)-3-buten-1-ol** [50]: ^1^H NMR (300 MHz, CDCl_3_) 7.43 (d, *J* = 8.58, 2H), 7.18 (d, *J* = 8.25, 2H), 5.80–5.65 (m, 1H), 5.13–5.07 (m, 2H), 4.64 (t, *J* = 6.51, 1H), 2.43 (t, *J* = 7.2, 2H).

**4-(1-Hydroxybut-3-enyl)benzonitrile** [51]: ^1^H NMR (500 MHz, CDCl_3_) 7.59 (d, *J* = 8.6, 2H), 7.44 (d, *J* = 7.45, 2H), 5.78–5.70 (m, 1H), 5.14–5.10 (m, 2H), 4.76 (t, *J* = 2.85, 1H), 2.48–2.41 (m, 2H).

**1-Cyclohexyl-3-buten-1-ol** [39]: ^1^H NMR (300 MHz, CDCl_3_) 6.0–5.7 (m, 1H), 5.2–5.0 (m, 1H), 3.5–3.3 (m, 1H), 2.4–2.2 (m, 1H), 2.1–2.0 (m, 1H), 1.9–1.6 (m, 4H), 1.5–1.0 (m, 4H).

**1-(4-Methylphenyl)-3-buten-1-ol** [39]: ^1^H NMR (300 MHz, CDCl_3_) 7.24 (d, *J* = 8.25, 2H), 7.16 (d, *J* = 8.22, 2H), 5.87–5.73 (m, 1H), 5.17–5.10 (m, 2H), 4.89 (s, 1H), 4.69 (t, *J* = 6.54, 1H), 2.51 (t, *J* = 6.6, 2H), 2.35 (s, 3H).

**1-(3-Methylphenyl)-3-buten-1-ol** [50]: ^1^H NMR (500 MHz, CDCl_3_) 7.24 (t, *J* = 7.45, 1H), 7.18 (s, 1H), 7.14 (d, *J* = 8.05, 1H), 7.09 (d, *J* = 7.45, 1H), 5.85–5.77 (m, 1H), 5.18–5.12 (m, 2H), 4.69 (t, *J* = 5.15, 1H), 2.50 (t, *J* = 7.45, 2H), 2.36 (s, 3H).

**1-(2-Methylphenyl)-3-buten-1-ol** [50]: ^1^H NMR (300 MHz, CDCl_3_) 7.27–7.14 (m, 4H), 5.91–5.80 (m, 1H), 5.22–5.14 (m, 2H), 4.97 (t, *J* = 3.6, 1H), 2.49 (t, *J* = 8.25, 2H), 2.34 (s, 3H).

**1-Phenyl-3-buten-1-ol** [39]: ^1^H NMR (300 MHz, CDCl_3_) 7.38–7.35 (m, 3H), 5.85–5.76 (m, 2H), 5.15–5.12 (m, 2H), 4.73 (t, *J* = 1.38, 1H), 2.51 (t, *J* = 1.02, 2H).

**1-Phenyl-1,5-hexadien-3-ol** [52]: ^1^H NMR (300 MHz, CDCl_3_) 7.41–7.22 (m, 5H), 6.61 (d, *J* = 16.5, 1H), 6.25 (dd, = 6.54, 15.78 Hz, 1H), 5.90–5.79 (m, 1H), 5.22–5.15 (m, 2H), 4.36 (q, *J* = 5.85, 1H), 2.41 (q, *J* = 8.94, 2H).

## Data Availability

The data that supports the findings of this study is available from the corresponding author upon reasonable request.

## References

[1] Yan M, Kawamata Y, Baran P S (2017). Chem Rev.

[2] Nozaki S, Suzuki Y, Goto T (2024). Electrochim Acta.

[3] Kong X, Liu Q, Chen Y, Wang W, Chen H-F, Wang W, Zhang S, Chen X, Cao Z-Y (2024). Green Chem.

[4] David M, Galli E, Brown R C D, Feroci M, Vetica F, Bortolami M (2023). Beilstein J Org Chem.

[5] Witherspoon E, Ling P, Winchester W, Zhao Q, Ibrahim A, Riley K E, Wang Z (2022). ACS Omega.

[6] Rocco D, Chiarotto I, Mattiello L, Pandolfi F, Zane D, Feroci M (2019). Pure Appl Chem.

[7] Yimin D, Lanli N, Hui L, Jiaqi Z, Linping Y, Qiuju F (2018). Int J Electrochem Sci.

[8] Kathiresan M, Velayutham D (2015). Chem Commun.

[9] Zhao S-F, Horne M, Bond A M, Zhang J (2015). Phys Chem Chem Phys.

[10] Kronenwetter H, Husek J, Etz B, Jones A, Manchanayakage R (2014). Green Chem.

[11] Orsini M, Chiarotto I, Feeney M M M, Feroci M, Sotgiu G, Inesi A (2011). Electrochem Commun.

[12] Liu Y-Z, Lin M-Y, Xiao L-P, Zhang K, Lu J-X (2008). Chin J Chem.

[13] Mellah M, Zeitouny J, Gmouh S, Vaultier M, Jouikov V (2005). Electrochem Commun.

[14] Doherty A P, Brooks C A (2004). Electrochim Acta.

[15] Mellah M, Gmouh S, Vaultier M, Jouikov V (2003). Electrochem Commun.

[16] Bornemann S, Handy S T (2011). Molecules.

[17] Smith E L, Abbott A P, Ryder K S (2014). Chem Rev.

[18] Hansen B B, Spittle S, Chen B, Poe D, Zhang Y, Klein J M, Horton A, Adhikari L, Zelovich T, Doherty B W (2021). Chem Rev.

[19] Protsenko V (2024). Coatings.

[20] Mousa M O, Adly M E, Mahmoud A M, El-Nassan H B (2024). ACS Omega.

[21] El-Nassan H B, El-Mosallamy S S, Mahmoud A M (2023). Sustainable Chem Pharm.

[22] Osman E O, Mahmoud A M, El-Mosallamy S S, El-Nassan H B (2022). J Electroanal Chem.

[23] Trujillo S A, Peña-Solórzano D, Bejarano O R, Ochoa-Puentes C (2020). RSC Adv.

[24] Golgovici F, Anicai L, Florea A, Visan T (2020). Curr Nanosci.

[25] Yamamoto Y, Asao N (1993). Chem Rev.

[26] Denmark S E, Fu J (2003). Chem Rev.

[27] Gonzalez-Gallardo N, Saavedra B, Guillena G, Ramón D J (2021). Appl Organomet Chem.

[28] Yusof R, Abdulmalek E, Sirat K, Rahman M B A (2014). Molecules.

[29] Zhang J, Zhang L, Xie W, Chen M, Zhang C, Qin Y, Zhao J, Wang F, Liu Z-Q (2024). Green Chem.

[30] Zhang Q, Liang K, Guo C (2022). Angew Chem, Int Ed.

[31] Torabi S, Jamshidi M, Amooshahi P, Mehrdadian M, Khazalpour S (2020). New J Chem.

[32] Sinha A K, Mondal B, Kundu M, Chakraborty B, Roy U K (2014). Org Chem Front.

[33] Sun L, Sahloul K, Mellah M (2013). ACS Catal.

[34] de Souza R F M, Areias M C C, Bieber L W, Navarro M (2011). Green Chem.

[35] Zhang L, Zha Z, Zhang Z, Li Y, Wang Z (2010). Chem Commun.

[36] Huang J-M, Ren H-R (2010). Chem Commun.

[37] Zhang L, Zha Z, Wang Z, Fu S (2010). Tetrahedron Lett.

[38] Huang J-M, Dong Y (2009). Chem Commun.

[39] Zha Z, Hui A, Zhou Y, Miao Q, Wang Z, Zhang H (2005). Org Lett.

[40] Durandetti M, Meignein C, Périchon J (2003). J Org Chem.

[41] Hilt G, Smolko K I (2001). Angew Chem, Int Ed.

[42] Rollin Y, Derien S, Duñach E, Gebehenne C, Perichon J (1993). Tetrahedron.

[43] Hebri H, Duñach E, Périchon J (1993). Tetrahedron Lett.

[44] Tokuda M, Uchida M, Katoh Y, Suginome H (1990). Chem Lett.

[45] Tokuda M, Satoh S, Suginome H (1989). J Org Chem.

[46] Durandetti S, Sibille S, Perichon J (1989). J Org Chem.

[47] Minato M, Tsuji J (1988). Chem Lett.

[48] Uneyama K, Matsuda H, Torii S (1984). Tetrahedron Lett.

[49] Lapeña D, Lomba L, Artal M, Lafuente C, Giner B (2019). Fluid Phase Equilib.

[50] Niharika P, Ramulu B V, Satyanarayana G (2018). ACS Omega.

[51] Dam J H, Fristrup P, Madsen R (2008). J Org Chem.

[52] Smith K, Lock S, El-Hiti G A, Wada M, Miyoshi N (2004). Org Biomol Chem.

